# Indoor Air Pollution and Delayed Measles Vaccination Increase the Risk of Severe Pneumonia in Children: Results from a Case-Control Study in Mwanza, Tanzania

**DOI:** 10.1371/journal.pone.0160804

**Published:** 2016-08-10

**Authors:** George PrayGod, Crispin Mukerebe, Ruth Magawa, Kidola Jeremiah, M. Estée Török

**Affiliations:** 1 Mwanza Medical Research Centre, National Institute for Medical Research, Mwanza, Tanzania; 2 Department of Medicine, University of Cambridge, Cambridge, United Kingdom; Universite de Nantes, FRANCE

## Abstract

**Background:**

Mortality due to severe pneumonia during childhood in resource-constrained settings is high, but data to provide basis for interventions to improve survival are limited. The objective of this study was to determine the risk factors for severe pneumonia in children aged under five years old in Mwanza, Tanzania.

**Methods:**

We conducted a case-control study of children aged 2 to 59 months at Sekou-Toure regional hospital in Mwanza City, north-western, Tanzania from May 2013 to March 2014. Cases were children with severe pneumonia and controls were children with other illnesses. Data on demography, social-economical status, nutritional status, environmental factors, vaccination status, vitamin A supplementation and deworming, and nasopharyngeal carriage were collected and analysed using logistic regression.

**Results:**

117 patients were included in the study. Of these, 45 were cases and 72 controls. Cases were younger than controls, but there were no differences in social-economic or nutritional status between the two groups. In multiple regression, we found that an increased risk of severe pneumonia was associated with cooking indoors (OR 5.5, 95% CI: 1.4, 22.1), and delayed measles vaccination (OR 3.9, 95% CI: 1.1, 14.8). The lack of vitamin A supplementation in the preceding six month and *Enterobacter* spp nasopharyngeal carriage were not associated with higher risk of severe pneumonia. Age ≥24 months (OR 0.2, 95% CI: 0.04, 0.8) and not receiving antibiotics before referral (OR 0.3, 95% CI 0.1, 0.9) were associated with lower risk for severe pneumonia.

**Conclusions:**

Indoor air pollution and delayed measles vaccination increase the risk for severe pneumonia among children aged below five years. Interventions to reduce indoor air pollution and to promote timely administration of measles vaccination are urgently needed to reduce the burden of severe pneumonia in children in Tanzania

## Background

Pneumonia is responsible for 18% of nearly 7.6 million deaths in children aged under five globally[1]. Efforts to improve childhood survival have included increasing access to vaccines against *Streptococcus pneumoniae* and *Haemophilus influenzae*, but these have not yet shown a major impact[1, 2]. This may suggest that that pneumonia-related deaths may be caused by other bacteria and viruses for which there are no already existing vaccines [3]. Although these other pathogens are necessary factors for severe pneumonia, they are not necessarily sufficient cause for severe pneumonia. Other attributes, including host and environmental factors are needed to interact with these pathogens to produce sufficient causal conditions for the development of the disease[4]. There is relatively little data on these other factors (risk factors) which are important for completion of causation process and their relative importance in the occurrence of severe pneumonia during childhood in our setting.

In India and the Gambia, environmental pollution, and malnutrition were the major risk factors for severe pneumonia-and pneumonia-related mortality [5, 6], whereas studies in South Africa and Mozambique suggested that demographic, bacterial virulence, and malnutrition increased the risk of poor pneumonia treatment outcomes [7, 8]. These studies suggest that there may be regional differences in the relative importance of risk factors for childhood severe pneumonia, although data are limited and inconsistent. This information is urgently needed in order to be able to provide evidence base for testing, and adapting interventions which will reduce severe pneumonia during childhood, particularly in low-income settings where the burden is highest and contribute in achieving sustainable development goals.

We conducted a case-control study to assess the role of demographic, environmental and nutritional correlates, and nasopharyngeal bacterial carriage as risk factors for severe pneumonia among children aged below five years in Mwanza, Tanzania.

## Methods

### Study design and population

We conducted a prospective case-control study to assess risk factors for severe pneumonia among children aged under five in Mwanza city, in north-western, Tanzania. Children aged 2–59 months attending Sekou-Toure hospital between May 2013 and March 2014 were eligible for inclusion in the study. Children who were diagnosed with either severe pneumonia or very severe pneumonia based on World Health Organization definitions[9] at admission, who were about to receive, but had not received any treatment for severe pneumonia or very severe pneumonia at the hospital were defined as cases. Children presenting with cough or difficulty in breathing were diagnosed with severe pneumonia if they had fast breathing (≥50 breaths per minute for those aged 2 to 11 months and ≥40 breaths for those aged 12 to 59 months) and at least one of the following: lower chest indrawing, nasal flaring or grunting. Children were diagnosed with very severe pneumonia if they had above clinical presentations for severe pneumonia and danger signs which included any of the following: central cyanosis, severe respiratory distress, and inability to breastfeed or drink, vomiting everything, and convulsions, lethargy or unconsciousness. Children aged 2 to 59 years who were being attended at the outpatient department or admitted into the hospital pediatric ward during the same day as cases who were diagnosed with illnesses other than pneumonia, severe pneumonia, or very severe pneumonia were included as controls. As for the cases, diagnosis of illnesses in the controls followed WHO guidelines[9]. Children were excluded if they had other severe illnesses including cardiac or renal diseases. Study clinicians and nurses were thoroughly trained on the study protocol, but were not made aware of the study hypotheses. Similarly, parents and guardians of recruited children were made aware of the study objectives but not to specific hypotheses being explored.

### Sample size and power calculation

Based on previous studies we made an assumption that indoor air pollution (including indoor cooking) would be a major risk factor for severe pneumonia among children aged <5 years [10, 11]. We hypothesized that the prevalence of exposure (indoor air pollution) among the controls would be around 15%[12], and the exposure would increase the risk of severe pneumonia by a factor of four or more [10]. Thus, we estimated that we would require 42 patients in each group (cases and controls) to detect this risk with 80% power at 5% significant level[13].

### Data collection

We collected demographic data, vaccination history and socio-economic status (SES) by interviewing the guardian or parent using structured questionnaires, and by retrieving information from the participant’s case files and postnatal clinic card. We collected SES data using proxy indicators such as possession of household items e.g. sofa, radio, fridge, wall clock, television and bicycle [14]. Based on this, we defined possession of two assets or less as low SES, of three or four assets as medium SES and of five or six assets as high SES. We also collected information on education level of parents, and used these as further proxy for SES. To assess if a crowded environment was a risk factor for severe pneumonia, we asked interviewees to give information on the number of rooms in their household, and number of people sleeping in each room. We then divided the number of rooms by the number of people sleeping in each room to get an indication of crowdedness in the households. We also collected data on the source of cooking fuel (electricity, gas, wood charcoal, or firewood) and asked if the preparation of household food occurred indoors or outdoors, to ascertain the role of indoor air pollution as risk factor for severe pneumonia. To further assess the role of pollution, we asked whether either parent was a regular smoker. Parents/guardians provided post-natal clinic cards to ascertain whether study participants had received measles vaccination and were asked whether their children had received vitamin A supplementation, and antihelminthic drugs in the previous six months. We also collected information on whether patients received treatment within 24 hours or five days of onset of illness and whether the treatment given before referral was an antibiotic. Weight was collected using standard methods and used to calculate weight-for-age *z*-scores on the basis of WHO 2006 growth standards[15]. A child was defined as underweight if his/her weight-for-age *z*-score was less than –2 z score, and as severely underweight if his/her weight-for-age *z*-score was less than –3 z score. Children with >-2 z score were considered to be normal.

### Collection and processing of nasopharyngeal swabs

Because of the difficult in collecting specimens from lower respiratory tract to establish bacterial aetiologies for severe pneumonia, we opted to collect nasopharyngeal swabs as a proxy for lower respiratory tract specimens. The swabs were collected from cases and controls using standard methods [16, 17], and after collection they were transferred to National Institute for Medical Research (NIMR) Microbiology Laboratory for processing and were sub-cultured on blood, MacConkey and chocolate agar. For positive nasopharyngeal cultures, the isolates were identified using Gram staining and standard identification methods [18] and assessed as potential risk factors for severe pneumonia.

### Statistical analysis

Data were collected in individual case record forms and double entered in EpiData (EpiData Association, Odense, Denmark) and analyzed using Stata/IC version 12.0 (StataCorp LP, College Station, TX, USA). Normal probability plots were used to assess normality of continuous variables. Differences in categorical and continuous variables between groups were tested using chi-squared test and t-test respectively. Potential risk factors for severe pneumonia included age, gender, SES, nutritional status, measles vaccination, vitamin A supplementation, deworming, environmental factors (e.g. indoor use of unprocessed biomass fuel and crowding), parental smoking, health system factors, and nasopharyngeal bacterial carriage were initially assessed using univariate analysis. Those whose effect sizes were significant at *P*<0.15 were included in the final multiple logistic regression to control for multiple confounding and to test for interactions between predictor variables. Likelihood ratio test was used to measure goodness of the fit of the multiple regression models. Age was included in models as categorical (<2yrs/≥2years) rather than continuous, since children in these age groups may have different disease risk due to differing immunity levels. The effect sizes were presented as odds ratios (OR) with 95% confidence intervals. *P* values <0.05 indicated significant difference.

### Ethical considerations

Written informed consent for participation in the study was obtained from parents or guardians of children recruited to the study and followed principles laid down in the Declaration of Helsink. The study received ethical approval from the National Medical Research Coordinating Committee of the National Institute for Medical Research (NIMR) in Dar es Salaam, in Tanzania.

## Results

### Background characteristics

Data for 117 patients were available. Of these children, 55 (47%) were females, the mean age was 22.1 (SD 16.2) months, and 45 were cases with severe pneumonia, and 72 were controls. Of the children recruited as controls, 55 (76.4%) had malaria, 11(22.2) had diarrhea, 3 (4.2%) had anaemia, 1 (1.4%) had urinary tract infection, 1 (1.4%) had lymphadenopathy, and the remaining 1 (1.4%) had sickle cell disease. Children in the severe pneumonia group were younger (16 months *versus* 26 months, *P* = 0.001) and had longer duration of illness (10.5 days *versus* 5.4 days, *P* = 0.03) compared with those in the control group, but there were no differences in gender, SES, and nutritional status distribution between the two groups (Table 1). There were, however, significant differences in mother’s marital status between the two groups (*P* = 0.04). Cases were more likely to be treated with antibiotics before referral (82.4% *versus* 40%, *P* = 0.01) than controls, but they were less likely to have received measles vaccination (54% *versus* 90%, *P* = 0.0001) as shown in Table 2. We further explored the patterns of measles vaccination and found most of the difference between cases and controls was mainly explained by difference in coverage among children aged 0–12 months where much more controls received vaccination than cases (27.3% versus 64.3%, *P* = 0.03), since comparison in other age groups between cases and controls were not statistically significant. Although a smaller proportion of cases had received vitamin A supplementation in the past 6 months compared to controls, this did not reach statistical significance (6.7% versus 16.7%, *P* = 0.16).

**Table 1:** Comparison of baseline characteristics of 117 patients recruited into the study as cases and controls^1^.

	Cases (n = 45) ^2^	Controls (n = 72)^3^	*P*^4^
Age (months)	16.1 (±14.1)	26.1 (±16.4)	0.001
2–23 months	36 (80)	40 (55.6)	0.007
24–59 months	9 (20)	32 (44.4)	
Gender			
Females	22 (48.9)	33 (45.8)	0.75
Males	23 (51.1)	39 (54.2)	
Duration of illness (days)	10.8 ±19.1	5.4 ±6.7	0.03
Mother’s marital status			
Married/cohabiting	44 (100)	66 (91.7)	0.04
Widow/divorced/single	0	6 (8.3)	
Mother’s religion			
None	0	3 (4.2)	0.36
Christian	34 (75.6)	50 (69.4)	
Muslim	11 (24.4)	19 (29.4)	
Mother’s education level			
Never attended school	5 (11.1)	8 (11.1)	0.71
Primary education	27 (60.0)	48 (66.7)	
Secondary/college education	13 (28.9)	16 (22.2)	
Socio-economic status (SES)^5^			
Low	19 (42.2)	43 (59.7)	0.17
Middle	16 (35.6)	19 (26.4)	
High	10 (22.2)	10 (13.9)	
Nutritional status			
Normal weight	36 (80.0)	51 (70.8)	0.15
Moderate underweight	3 (6.7)	14 (19.4)	
Severe underweight	6 (13.3)	7 (9.7)	

^1^Data are in mean±SD or n (%)

^2^Due to missing values some variables do not add up to 45

^3^Due to missing values some variables do not add up to 72

^4^Comparison between cases with controls using ttest or chi-square

^5^Assessed based on possession of fridge, sofa, bicycle, television, radio and wall clock; possession of up 2 assets was regarded as low SES, possession of 2 to 4 assets was regarded as middle SES, and possession of 5 to 6 assets was regarded as high SES

**Table 2:** Clinical characteristics of 117 patients recruited into the study as cases and controls^1^^,^ ^2^.

	Cases (n = 45) ^2^	Controls (n = 72)^3^	*P*^4^
Received any treatment within 24 hours			
Yes	17 (40.5)	25 (36.8)	0.70
No	25 (59.5)	43 (63.2)	
Treated with any antibiotic before referral			
Yes	24 (75)	22 (36.1)	0.0001
No	8 (25)	39 (63.9)	
Measles vaccination			
Yes	22 (53.7)	63 (90)	<0.0001
No	19 (46.3)	7 (10)	
Vitamin A supplementation in past 6 months			
Yes	3 (6.7)	12 (16.7)	0.16
No	42 (93.3)	60 (83.3)	
Deworming in past 6 months (age>13 months)			
Yes	3 (15.8)	9 (16.1)	0.98
No	6 (84.2)	47 (83.9)	
*Streptococcus pneumoniae*			
Yes	7 (15.6)	5 (7.0)	0.21
No	38 (84.4)	66 (93.0)	
*Klebsiella pneumoniae*			
Yes	2 (4.4)	2 (2.8)	0.64
No	43 (95.6)	69 (97.2)	
*Escherichia coli*			
Yes	1 (2.2)	0 (0)	0.39
No	44 (97.8)	71 (100)	
*Streptococcus pyogenes*			
Yes	1 (2.2)	1 (1.4)	1.00
No	44 (97.8)	70 (98.6)	
*Enterobacter* spp			
Yes	9 (20)	3 (4.2)	0.01
No	36 (80)	68 (95.8)	

^1^Data are in mean±SD or n (%)

^2^Due to missing values some variables do not add up to 45

^3^Due to missing values some variables do not add up to 72

^4^Comparison between cases with controls using ttest or chi-square test.

Regarding nasopharyngeal carriage, bacteria were isolated in 24% (28/117) of study participants and they included *Streptococcus pneumonia*, *Klebsiella pneumoniae*, *Escherichia coli*, *Streptococcus pyogenes*, *and Enterobacter* spp. As seen in Table 2, there were more cases with *Enterobacter* spp than controls, but there was no difference in other isolates between the two groups.

### Risk factors for severe pneumonia

To further understand risk factors for severe pneumonia, we conducted univariate and later multiple logistic regression analyses. In univariate analyses comparing cases with controls, we found that cooking indoors, delayed measles vaccination, and nasopharyngeal colonization with *Enterobacter* spp increased the risk of severe pneumonia, but age ≥24 months and not receiving antibiotics before referral were protective against severe pneumonia (Table 3). We then included correlates with *P*<0.15 in univariate analyses into a multivariable model to control for confounding. In this model, we found that cooking indoors (OR 5.5, 95% CI:1.4, 22.1), and delayed measles vaccination (OR 3.9, 95% CI: 1.1, 14.8) were significant risk factors for severe pneumonia. Lack of vitamin A supplementation in the preceding 6 months (OR 6.1, 95% CI: 0.5, 67.4) and *Enterobacter* spp carriage (OR 4.1, 95% CI 0.6, 26.7) were associated with higher risk of severe pneumonia, but these associations did not reach statistical significance level (Table 4). In addition, we found that age above ≥24 months (OR 0.2, 95% CI: 0.04, 0.8) and not receiving antibiotics before referral (OR 0.3, 95% CI: 0.1, 0.9) were associated with reduced risk of having severe pneumonia.

**Table 3:** Univariate logistic regression analysis of risk factors for severe pneumonia among children aged below five years in Mwanza.

	Controls	Cases	Odds Ratio (95% CI)	*P* value
	N (%)	N (%)		
Age (months)				
2–23	40 (56)	36 (80)	Reference	
24–59	32 (44)	9 (20)	0.31 (0.1, 0.7)	0.008
Sex				
Females	33 (46)	22 (49)	Reference	
Males	39 (54)	23 (51)	0.89 (0.4, 1.9)	0.75
Source of cooking fuel				
Gas/electricity	3 (4.2)	1 (2.2)	Reference	
Firewood	7 (9.9)	5 (11.4)	2.1 (0.2, 27)	0.84
Wood charcoal	61 (85.9)	38 (86.4)	1.9 (0.2, 18)	
Location of kitchen				
Cooking outdoor	48 (70.6)	23 (52.3)	Reference	
Cooking indoor	20 (29.4)	21 (47.7)	2.2 (0.99, 4.8)	0.05
Smoking status				
Smoking	3 (4.2)	3 (6.7)	Reference	
Non-smoking	68 (95.8)	42 (93.3)	0.6 (0.1, 3.2)	0.57
Number of people per sleeping room	1.9	2.0	1.1 (0.7, 1.7)	0.62
Socio-economic status (SES)^1^				
Low	43 (59.7)	19 (42.2)	Reference	
Middle	19 (26.4)	16 (35.6)	1.9 (0.8, 4.5)	0.18
High	10 (13.9)	10 (22.2)	2.3 (0.8, 6.3)	
Measles vaccination			8.1 (2.2, 29.8)	
Vaccinated	63 (90)	22 (53.7)	Reference	
Not vaccinated	7 (10)	19 (46.3)	7.8 (2.9, 20.9)	0.0001
Vitamin A supplementation in the past 6 months				
Supplemented	12 (16.7)	3 (6.7)	Reference	
Not supplemented	60 (83.3)	42 (93.3)	2.8 (0.7, 10.5)	0.13
Dewormed in the past 6 months				
Dewormed	9 (12.5)	5 (11.1)	Reference	
Not dewormed	63 (87.5)	40 (88.9)	1.4 (0.4, 3.7)	0.82
Received treatment within 24 hours				
Yes	25 (36.8)	17 (40.5)	Reference	
No	43 (63.2)	25 (59.5)	0.9 (0.4, 1.9)	0.70
Received antibiotics before referral				
Yes	22 (36.1)	24 (75)	Reference	
No	39 (63.9)	8 (25)	0.2 (0.07, 0.5)	0.0006
Nutritional status				
Normal weight	51 (70.8)	36 (80)	Reference	
Moderate underweight	14 (19.4)	3 (6.7)	0.3 (0.08, 1.3)	0.18
Severe underweight	7 (9.8)	6 (13.3)	1.2 (0.4, 3.9)	
*Streptococcus pneumoniae*				
Not isolated	66 (93)	38 (84.4)	Reference	
Isolated	5 (7)	7 (15.6)	2.4 (0.7, 8.2)	0.15
*Klebsiella pneumoniae*				
Not isolated	69 (97.2)	43 (95.6)	Reference	
Isolated	2 (2.8)	2 (4.4)	1.6 (0.2, 11.8)	0.64
*Streptococcus pyogenes*				
Not isolated	70 (98.6)	44 (97.8)	Reference	
Isolated	1 (1.4)	1 (2.2)	1.6 (0.1, 26.1)	0.75
*Enterobacter* spp				
Not isolated	68 (95.8)	36 (80)	Reference	
Isolated	3 (4.2)	9 (20)	5.7 (1.4, 22.2)	0.01

^1^Assessed based on possession of fridge, sofa, bicycle, television, radio and wall clock; possession of up 2 assets was regarded as low SES, possession of 2 to 4 assets was regarded as middle SES, and possession of 5 to 6 assets was regarded as high SES

**Table 4:** Multiple logistic regression analysis of risk factors for severe pneumonia among children aged below five years in Mwanza^1^.

	Odds Ratio (95% CI)	*P* value
Age (months)		
2–23	Reference	
24–59	0.2 (0.04, 0.8)	0.02
Sex		
Females	Reference	
Males	1.0 (0.3, 3.4)	0.94
Location of kitchen		
Cooking outdoor	Reference	
Cooking indoor	5.5 (1.4, 22.1)	0.02
Measles vaccination		
Vaccinated	Reference	
Not vaccinated	3.9 (1.1, 14.8)	0.04
Vitamin A supplementation in the past 6 months		
Supplemented	Reference	
Not supplemented	6.1 (0.5, 67.4)	0.14
Received antibiotics before referral		
Yes	Reference	
No	0.3 (0.1, 0.9)	0.04
*Enterobacter* spp		
Not isolated	Reference	
Isolated	4.1 (0.6, 26.7)	0.14

^1^variables in the model were adjusted for each other

## Discussion

Despite increased access to vaccines against major causes of acute respiratory tract infections, pneumonia remains a major cause of child morbidity and mortality, causing about 1.4 million deaths annually [19], most of them from low-and middle-income countries (LMIC). In Tanzania for example, although *Haemophilus influenza* and pneumococcal vaccines were introduced in 2009 and 2013 respectively, data suggest that proportions of children with pneumonia-related signs seen in healthcare settings and those reported in population surveys have not changed after introduction of these vaccines[20–22]. Thus, in addition to deployment of vaccines, work is needed to further understand determinants of severe pneumonia, which is the ultimate reason for death among children with pneumonia. In the present study, we report that the delay of measles vaccination and cooking indoor increase the risk of severe pneumonia among under-fives whereas age ≥2 years and not receiving antibiotics during the first 24 hours of onset of illness were associated with reduced risk for severe pneumonia. In this study we isolated 5 nasopharyngeal bacteria, but all including *Streptococcus pneumonia and Enterobacter* spp, the most frequent isolated bacteria in our participants were not related to an increase risk for severe pneumonia.

According to the World Health Assembly [23], measles vaccination coverage among children aged 1 year should be ≥80% for this vaccine to have effects on measles transmission. In this study we report measles vaccination coverage of 53.7% among cases compared to 90% among the controls, explaining about four-fold increased risk for severe pneumonia among cases compared to controls.

However, considering that this was a hospital rather a community-based case-control study, the risk estimates for severe pneumonia are likely to have been underestimated, since hospital based controls are likely to be similar to cases [24]. On further exploration of measles immunization coverage, we found that the lower vaccination coverage in the cases compared to controls was mainly confined in children aged ≤1 year. In fact, this difference in coverage waned off as the children age increased and by the end of second year, it was no longer statistically significant between the two groups. This indicates that late rather than no vaccination led to a higher risk of severe pneumonia and that increasing measles vaccination coverage before the end of first year of life could reduce the risk of severe pneumonia in this age group. Since children with measles are known to have higher risk for development of pneumonia, this association may imply the possibility of having a measles outbreak in the study area during the study period. However, this is unlikely considering that such an outbreak would have been detected through ongoing childhood illnesses surveillance system conducted by the Ministry of Health. Another possible explanation is that measles vaccination reduces the risk of infections including pneumonia causing organisms probably by enhancing immunity [25]. This concurs with previous work where investigators demonstrated that measles vaccine may have other beneficial effects in addition to conferring immunity to measles [26]. These data suggest that early and timely vaccination against measles could contribute to prevention of severe pneumonia during childhood in our setting. The Ministry of Health and Social Welfare efforts to increase immunity against measles during childhood[27], should be extended to involve strategies to ensure timely administration of first dose of measles vaccine (at 9 month) to prevent occurrence of measles as well as severe pneumonia.

In the present study, we found that about 96.5% of cases and controls households were using unclean biomass fuel for cooking and that households with children with severe pneumonia had higher prevalence of using these forms of fuel indoor, which explained about five-fold increased risk for severe pneumonia in these households. Although the 95 CI was wide, this finding is similar to those from other studies that have documented increased risk of pneumonia among children who are exposed to air pollution[11], and suggests that a high proportion of severe pneumonia cases could be prevented by reducing indoor air pollution in this setting. Indoor air pollution could increase the risk for severe pneumonia by enhancing infection process by pneumonia pathogens. This may occur if exposure to pollutants contained in the biomass fuel either enhances attack of epithelia cells of respiratory tract and/or reduces specific and non-specific host immunity[28]. Irrespective of mechanisms explaining these relations, reduction of indoor air pollution will help reduce the risk in these populations and need to be promoted. However, currently, promotion of use of improved cooking stove and avoidance of indoor air pollution have not been given appropriate emphasis as a prevention measure for pneumonia, at least in SSA. To enhance the use of this approach, intervention studies testing locally acceptable strategies to reduce indoor air pollution are needed to reduce the burden of pneumonia in these settings. This should ensure that such tools are affordable to enhance sustainability in the resource-constrained settings.

In the present study we found that health system-related factors seemed to be important determinants of illnesses in our study participants. In both cases and controls, only about 40% of participants received any treatment within 24 hours of onset of illness, suggesting that two thirds of these children may have missed an opportunity to receive interventions before illnesses develop into severe forms. Patients with severe pneumonia were seen in the study hospital 10 days later after developing symptoms whereas as those in the control group were seen 5 days later after developing symptoms. This suggests that delay in seeking care could be one of the reasons explaining occurrence of severe pneumonia in this setting. Interestingly, in this study we found that children who had not received antibiotics before referral were at reduced rather than increased risk of developing severe pneumonia. Children who did not receive antibiotics before referral might be those who were less ill and therefore less likely to develop severe pneumonia whereas those who received antibiotics before referral were likely to be those with severe forms of illness. The fact that receipt of antibiotics did not prevent these children to develop severe pneumonia suggests that they were either inappropriate or ineffective. This suggests that the need to educate communities on the importance of timely care seeking in appropriate healthcare facilities. Further studies are needed to confirm these findings and to determine the optimal timing for treatment of community-acquired pneumonia in children aged under five in our setting.

Both cases and controls were recruited from children who were residents of Mwanza city who turned to be of the same socio-economical status and thus controls were representative of the population which produced cases. We trained study staff on diagnosis and management of common paediatric illnesses and parents and guardians of the recruited children were informed of the objectives of the study, but for both groups we did not disclose specific associations which were being explored. These design aspects reduced the likelihoodness of selection and information bias. Furthermore, age of a child and history of measles vaccination were ascertained using postnatal clinic cards, so it is highly unlikely that recall bias was introduced during collection of these exposure variables. Although information on cooking location was reported rather observed we see no possibility that recall bias was introduced considering that cooking is an ongoing daily activity that both cases and controls would need no effort to recall. However, regarding the use of antibiotics before referral, one cannot rule out that cases might have been able to remember the history of use than controls because of severity of the disease. However, since these controls were sick and not healthy such difference would be minimal considering that parents or guardians of controls would also want to bring all past exposure in full at the same level as cases to help understand the current illness. However, a small proportion of controls had diagnosis of diarrhoea. Diarrhoea may increase the risk of pneumonia[29, 30]. Therefore there is a possibility that inclusion of these patients reduced the strength of associations of the reported risk factors. However, data suggest that past history of diarrhoea (2–4 weeks) rather than current diarrhoea is the one associated with higher risk of pneumonia[29]. It is therefore unlikely that inclusion of children with a history of one or two days of diarrhoea would have biased the reported risk factors for severe pneumonia in this population. Overall we feel confident that our results were subject to little or no bias. However, further studies are needed to confirm these results and provide more background data to help address reported risk factors

## Conclusion

In conclusion, delayed measles vaccination and exposure to indoor air pollution increase the risk of severe pneumonia in Tanzania. To address this, the government ministries responsible for health, energy and environment need to urgently devise a research program for testing and adapting interventions for reduction of indoor air pollution such as switching from solid bio-fuel use to more cleaner and efficient energy alternatives (i.e. liquid petroleum gas, biogas, electricity, and solar power) in settings where this is feasible, using improved biomass cooking stoves, improving ventilation in cooking and living areas, and avoiding contact with smoke particularly by vulnerable groups like children. In addition, the expanded programme of immunization need to devise strategies for encouraging early and timely vaccination of children according to set schedules in order to reduce the risk of childhood diseases including pneumonia. Such strategies could involve, but not limited to regular public campaigns to increase vaccine awareness in communities as well as health education provided antenatally and postnatally. Exploration and ultimate adaptation of appropriate interventions will help reduce the burden of severe pneumonia and associated mortality in Tanzania
